# A Further Vindication of Lallemand

**Published:** 1882-01

**Authors:** Geo. H. Picard

**Affiliations:** Topeka, Kansas


### ARTICLE II.

### A FURTHER VINDICATION OF LALLEMAND. By GEO. H. PICARD, A.M., M.D., Topeka, Kansas.

Since the publication in the September number of THE JOURNAL AND EXAMINER of my paper, entitled "The Pathology of Involuntary Spermatic Fluxes," I have obtained a startling corroborative experience of the correctness of M. Lallemand's views on *diurnal seminal emissions.* It is the fashion among modern neurologists — who have developed a tendency to specialize spermatorhœa — either to ignore the manifestation of diurnal seminal losses occurring after defecation and micturition, or to ascribe their appearance to general plethora. According to Prof. Bartholow, they do not pathologically exist, and their presence is to be regarded as a physiological overflow. Prof. Austin Flint, Jr., less positively asserts that their reality is doubtful; and Prof. M. Rosenthal, even less radical, declines to discuss them.

These modern views of the neuro-spermatists—as I have already termed them—are not only an aggressive encroachment upon the rational theory of M. Lallemand and the Montpellier school, but are directly opposed to the well established theory and practice of the flower of British and Continental surgery. M. Lallemand, who instituted a series of cadaveric and clinical experimentation extending over a longer period and embracing a greater variety of cases than any subsequent observer, taught that diurnal involuntary spermatic fluxes were not uncommon sequences of prostatic irritation. Exactly how numerous these cases are, I am not prepared to affirm; suffice it to say, that within the last five years I have found a dozen of them, and I am satisfied that every one of them depended upon a structural modification of the prostatic urethra. That my cases were genuine spermatorrhœa of this variety, and that the lesions were purely local, and not prodromes or sequences of any ataxic condition, I desire to illustrate by giving the history of two of the more recent of them:

I. An Englishman, aged thirty-three; light complexion; pronouncedly lymphatic temperament; weight 220 lbs.; six years married; wife healthy; no children; temperate, highly intelligent; general health perfect; *a suo confessione* a devoted masturbater from early youth until his marriage; remembers to have had only two nocturnal emissions with an erection during his life; first noticed a passive discharge after defecation about two years before his marriage; consulted an eminent neurologist, who, without a microscopical examination, pronounced it a case of "prostatic sexual inquietude" of no importance, and declined to treat it. It is but just to add, that the existence of the habit was not revealed on that occasion. Had never essayed sexual intercourse previous to marriage; was unsuccessful in his first attempts at coition, and supposed himself impotent; after repeated trials, he succeeded in establishing intercourse, which he pursued with the assiduity he had given his old habit. The fluxes after defecation did not disappear, always returning on the third continent day; in his own words, "leaving me the choice between coition and emission." Becoming somewhat more abstinent after three years, the frequency of the discharges began to diminish, so that for the past year he has been able to remain continent for eight days, at the expiration of which they invariably return. The flow is perfectly passive and without sensation; his virile force seems undiminished, although his sexual apparatus shows signs of weakness, the penis being flabby, the testes somewhat too soft and shrunken, etc. He does not complain of any abatement of his sexual capacity.

My first impression, upon hearing this history, was a decided skepticism as to the seminal character of the discharge, and my unbelief was strengthened by the robust and cleanly appearance of the patient. After having several times microscopically examined the secretion, I became convinced of the truth of the story, having at each time found a genuine article of seminal fluid, containing an abundance of spermatozoa in full activity. After a most minute and diligent questioning, aided by the intelligent willingness of my patient, I learned that the fluxes were entirely passive, unaccompanied by the slightest sensation, and occurring at precisely the moment he arose from stool. He further stated that the flow was followed by no such feelings as are the sequences of coition—slight mental confusion, muscular relaxation, etc. Living for a considerable period *absque marito* made no perceptible improvement in matters. There was no existing constipation ; no evidence of any former urethral lesion ; no suspicion of stricture or blennorrhœa. I could not believe that the disorder was prodromatic of any ataxic trouble, both on account of its long continuance and the absence of anything like nervous debility in my patient. General plethora, as a causative factor, was excluded, from the fact that there was no continence. A somewhat curious feature of the case—and I believe I have noticed the same thing in all my cases—was the exact periodicity exhibited by the discharges, at first recurring after an interval of three continent days, the interval being lately increased to eight days. Concluding that the affection depended upon a chronic prostatic congestion, I injected, by means of the long-nozzled syringe, ʒ j of the tanno-glycerine, ʒ–℥, and enjoined perfect abstinence. After two weeks the flux returned. I then injected ʒ j of the strength of ʒ ij–℥j ; there was considerable discomfort and bloody urine for a day or two, but the flow has not returned, a period of nearly two months having already elapsed.

I hope that no one will consider that in this case I have mistaken prostatorrhœa for seminal loss. Although there was coexistent prostatic hyper-secretion, the microscope made the situation very clear.

II. This case is very similar in its general features to the preceding one, the chief points of difference being the less robust appearance of the patient, his having had gonorrhœa and balanitis before his marriage, and his less complete history of masturbation. Like number one, he complained of no loss of sexual power, no nocturnal emissions and no beginning of nervous disorder. Having been subject for several years to passive seminal losses after defecation, and although eight years married, still childless, he consulted me as to the probability of the seminal drain having affected his procreative power. The fluxes took place after a continence of four days. Believing a chronic prostatic hyperplasia to be the causative influence, I adopted a plan of treatment which I have since several times employed with the most satisfactory results, *the hot water irrigation of the urethra.* For this purpose I use a small-sized catheter having numerous lateral perforations and a bulbous tip, which will almost completely occlude the entrance to the bladder. To the catheter I attach a rubber tube, which is connected with an elevated vessel containing the water; the force of gravity does the rest, the water returning through the abundant space left in the urethra by the small catheter. I begin with the water at a temperature of 90° F., which I gradually increase to 115° or even 120° F. A daily sitting of ten or fifteen minutes will be found amply sufficient. Time and perseverance are necessary adjuncts to this treatment. I have found hot water to be decidedly more efficient in the treatment of chronic urethral congestion than the frigid hydro-therapeutics so highly recommended by the neurologists.

I am more than ever convinced of the truth of Lallemand's theory of *structural change.* I am delighted to see that the younger Gross, in his recent admirable work, has seen fit to check the engrossing ambition of the neurologists. The neuro-spermatists have furnished us a theory which is not justified by their practice, since they are compelled to rely altogether upon local measures. M. Trousseau adopted the rectum-pessary of a charlatan, and one of the most brilliant of living neurologists writes me "that for genuine passive spermatorrhœa, I know of nothing at all to be compared to the *porte caustique* of Lallemand."

I wish that morbid anatomists might continue the search for evidence of structural lesions in the urethræ of subjects known to have had spermatorrhœa; I cannot think they would be disappointed.

